# Anti-Obesity Effects of Soy Peptides In Vivo: A Meta-Analysis of Randomized Controlled Trials

**DOI:** 10.3390/foods15071191

**Published:** 2026-04-01

**Authors:** Zhendong Qiu, Zucai Wu, Keying Chen, Qiuyan Du, Yu Lin, Ming Li, Yuanhang Fan, Mengmeng Fan, Tingting Luo, Bo Song, Shanshan Liu

**Affiliations:** 1 Key Laboratory of Soybean Biology of the Chinese Education Ministry, Soybean Research Institute, Northeast Agricultural University, Harbin 150030, China; 2 Keshan Branch of Heilongjiang Academy of Agricultural Sciences, Qiqihar 161000, China; 3 Key Laboratory of Molecular and Cytogenetics, College of Life Sciences and Technology, Harbin Normal University, Harbin 150025, China

**Keywords:** soy peptide, anti-obesity, randomized controlled trial, meta-analysis, weight reduction

## Abstract

Soybean peptides have been implicated in reducing body weight (BW); however, their efficacy for weight loss remains ambiguous. This study aimed to evaluate the effects of soybean peptides on BW outcomes via meta-analysis. To identify randomized controlled trials (RCTs) from January 2000 to June 2025, numerous databases (PubMed, Embase, Web of Science, and Cochrane Library) were searched comprehensively. Studies comparing the impact of different soybean peptide interventions on weight reduction were included. The weighted mean difference (WMD) and 95% confidence interval (CI) were synthesized. Surface under the cumulative ranking curve (SUCRA) determined the intervention rankings, and 18 RCTs met the inclusion criteria. Overall, soybean peptides significantly decreased BW (WMD = −1.96 g, 95% CI: −3.52, −0.40; *p* < 0.00001), BW gain (BWG), and body mass index (BMI). Subgroup analyses confirmed lowering of BW in both humans (WMD = −12.71 g, 95% CI: −30.42, 5.00; *p* < 0.00001) and mice (WMD = −1.73 g, 95% CI: −3.44, −0.03; *p* < 0.00001). The above results indicate that soy peptides contribute significantly to weight loss.

## 1. Introduction

Obesity represents a chronic metabolic condition associated with elevated risk for several comorbidities, such as cardiovascular disorders [1], diabetes [2], and cancer [3,4,5,6]. Since 1990, data from the World Health Organization (WHO) have reflected a substantial rise in global obesity prevalence affecting both adults and children, with obesity rates tripling (adult) and quadrupling (childhood). Obesity (body mass index, BMI > 30) cannot be overlooked [7]; therefore, to mitigate its adverse health outcomes, various anti-obesity strategies have been adopted for weight loss, including dietary interventions and exercise-based regimens [8]. Balanced dietary intake is beneficial for weight control, and a wide range of edible substances exhibit anti-obesity properties. Edible plant extracts, namely eucalyptus leaf extract, *Opuntia stricta* var. *dillenii* fruit peel extract, and pickering emulsions collected from pomelo peel [9,10,11], exert anti-obesity effects. Likewise, certain edible microbes (*Clostridium butyricum B-3*, *Neoshirakia japonica* (Siebold & Zucc.) Esser [Euphorbiaceae], and *Bifidobacterium longum* subsp. longum HN001) have displayed anti-obesity activity [12,13,14]. In addition, a few natural compounds—ursolic acid, propolis, and human milk-derived 5′-UMP—have been shown to function as anti-obesity agents [15,16,17].

Soybeans are an important source of plant protein worldwide and possess potential disease-preventive properties [18,19,20]. Particularly beneficial for women, soy products can lower cholesterol levels as well as the risk of coronary artery disease, which contributes substantially to female mortality [21,22]. Evidence further suggests that soy consumption may help reduce the likelihood of multiple cancers (breast [20], endometrial [23], and prostate [24]), while improving bone health [25], cognitive function [26], and alleviating menopausal symptoms [27]. Apart from disease prevention, soy products play a key role in weight management [28]. Soy soluble polysaccharides promote weight loss by decreasing expression levels of lipid-binding proteins [29,30]. Soy β-conglycinin can minimize the incidence of fatty liver disease and help prevent obesity [31].

Previous studies put emphasis on the direct influence of soybean products on weight loss, with indicators involving body weight (BW), body fat, and other phenotypic traits [32,33,34]. However, the mechanisms underlying their anti-obesity effects warrant in-depth investigation. Soybean intake has been proposed to prevent obesity-related metabolic disorders in early life via modulating the gut microbiota, its metabolic profiles, and epigenetic programming in offspring [35]. Pharmacological analyses have identified key molecular targets of soybean products, including AKT1, SRC, STAT3, ESR1, FOS, and NFKB1, linked with the PI3K-Akt/JAK-STAT signaling axis. Soybean products interact strongly with these targets, causing a notable lowering of body weight gain (BWG), fat accumulation, and dyslipidemia. Concurrently, these molecules promote anti-obesity effects by reshaping the gut microbiome to alleviate oxidative stress and improve amino acid metabolism [36].

Despite numerous studies on various soy-derived components, the weight-reducing potential of soy peptides remains insufficiently explored, with divergent study designs and inconsistent findings [37]. Hence, an advanced analytical approach is required to systematically synthesize and analyze all available data on soy peptide-induced weight loss to draw a definitive conclusion. Meta-analysis enables the integration of direct comparisons (from head-to-head trials) and indirect evidence through common comparators, allowing concurrent assessment and hierarchy of different interventions within a single framework [38]. This analytic approach is fundamental for a systematic evaluation of soy peptides in weight regulation. To our knowledge, there is no meta-analytic report specifically focusing on the weight-loss effects of soybean peptides. Accordingly, this study aims to elucidate the impact of soy peptides on weight reduction and their relative contribution to BW reduction through meta-analysis.

## 2. Materials and Methods

### 2.1. Literature Survey

We searched the MEDLINE, PUBMED (National Library of Medicine, Bethesda, MD, USA), Web of Science, and the Cochrane Central Register of Controlled Trials (Clinical Trials; Central) from January 2000 to June 2025, using the following medical subject heading (MeSH) terms and keywords: soy, soybean, peptide, obesity, anti-obesity, overweight, weight loss, body weight, body fat, and BMI. Only studies involving human and animal subjects published in English-language journals were included. Reference lists of relevant original and review articles were manually screened. Two investigators independently examined the abstracts, keywords, or full-text reports initially retrieved from the search, dictating inclusion eligibility criteria. Any discrepancies regarding study selection were evaluated by additional investigators, and consensus was reached through discussion.

### 2.2. Study Selection Criteria

The following standards were used as inclusion criteria:

(I) The research subjects were animals or humans. A control and a treatment group were included, and an obesity model was established.
(II) The study design was a randomized controlled trial (RCT).
(III) The intervention group received treatment with a product containing soy peptides.
(IV) The control group received no treatment or was subjected to a vehicle intervention.
(V) Data for at least one obesity-related outcome measure (BW or BMI) were reported or could be calculated.

The exclusion criteria were as follows:

(I) Non-English publications, abstracts, letters, conference reports, or duplicate publications.
(II) Study participants with any prior condition known to affect obesity.
(III) The intervention was confounded by non-target components.
(IV) Co-interventions were present.
(V) Complete effect-size data could not be extracted or reliably derived from the study.

### 2.3. Data Extraction

According to the pre-defined inclusion and exclusion criteria, two investigators (Z.Q. and C.W.) independently conducted a preliminary screening of titles and abstracts, with subsequent full-text review to determine eligibility criteria for inclusion. Any disagreements in study selection were resolved by consulting a third investigator (T.L.). The same two investigators (Z.Q. and C.W.) independently extracted data following the Cochrane Handbook guidelines that included: author(s), year of publication, country, participant characteristics, intervention duration, sample size, treatment methods for intervention and control groups, and outcome measures (mean differences in BW and BWG, along with their standard deviations [SDs]). Data for which mean values or SDs could not be reliably obtained were excluded from the meta-analysis.

### 2.4. Risk of Bias Assessment

The assessment criteria encompassed seven sections: random sequence generation, allocation concealment, blinding of participants and personnel, blinding of outcome assessment, incomplete outcome data, selective reporting, and other bias. Each section was rated as “low risk,” “high risk,” or “some concerns”. The two investigators (Z.Q. and C.W.) independently evaluated the quality of the included literature. All discrepancies were addressed in discussion with the third investigator (T.L.) to reach an agreement.

### 2.5. Statistical Analysis

Two independent frequentist random-effects meta-analyses were conducted using Stata 18.0 (Stata Corp, College Station, TX, USA) and Review Manager 5.4 (The Cochrane Collaboration, Copenhagen, Denmark) software to determine the effect of soy peptides on BWG in humans and mice, respectively. The frequentist design was selected due to its easy implementation and comprehensive toolkit available within the Stata platform.

A significance threshold of *p* < 0.05 was applied, and the meta-analysis focused predominantly on BW, BWG, and BMI, according to the included literature. Other obesity-related indicators (e.g., blood-related parameters) were not incorporated in the meta-analysis because of the scarce availability of eligible studies or inadequately comparable interventions.

Since all outcomes were continuous variables, effect sizes were expressed as the mean difference ± SD between the intervention and control groups for each trial comparison. Sources of heterogeneity were examined through subgroup and sensitivity analyses. For each outcome supported by three or more studies, subgroup analysis was undertaken based on the study population (humans vs. mice). In case of significant local inconsistency observed in the subgroup analysis, sensitivity analysis was carried out by omitting studies that introduced inconsistency. Funnel plots combined with Egger’s test were applied to determine publication bias for outcomes involving more than 10 comparisons.

## 3. Results

### 3.1. Study Selection

**Figure 1:**
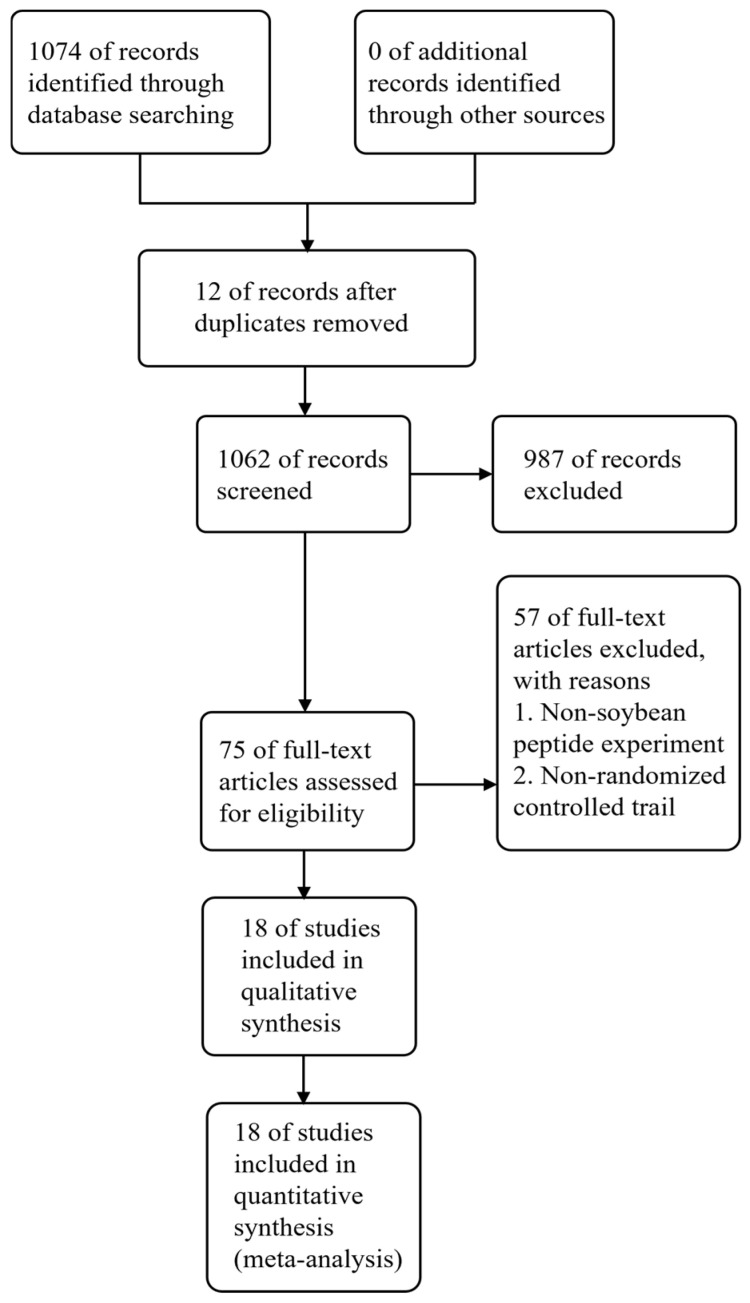
Schematic flowchart of the study selection process.

A total of 1074 records were initially identified through database searches. After removing 12 duplicate records, 1062 records were subjected to screening of titles and abstracts, further excluding 987 records that were deemed irrelevant. Full-text assessment of the remaining 75 articles resulted in the elimination of 57 that failed to fulfill the inclusion criteria. Ultimately, 18 studies were incorporated in the final analysis (Figure 1).

### 3.2. Study Characteristics

The specific characteristics of the 18 selected studies [39,40,41,42,43,44,45,46,47,48,49,50,51,52,53,54,55,56] are detailed in Table 1. Among obesity-related indicators, ten studies reported changes in BW, seven studies focused on BWG, and only one study investigated BMI. The intervention duration ranged from 2 to 26 weeks. In terms of study subjects, fifteen were rat-specific studies, while three studies conducted human interventions. Regarding their geographical distribution, two studies originated from Indonesia, seven from Japan, and nine from Republic of Korea. Concerning intervention materials, six studies utilized soy peptides, while twelve studies employed soy peptide-containing products.

### 3.3. Meta-Analysis and Subgroup Analysis

**Figure 2:**
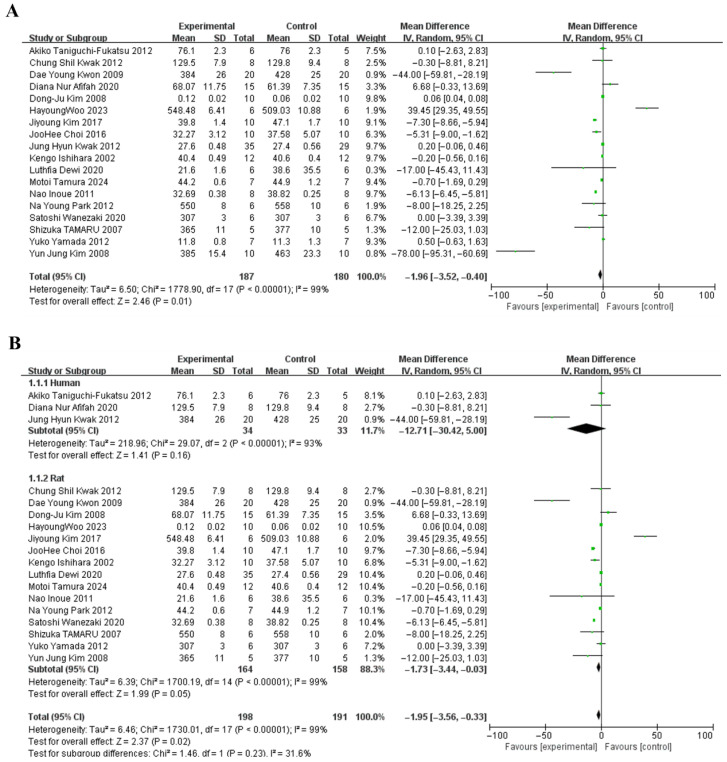
Forest plots showing (**A**): the effects of soy peptides on anti-obesity outcomes ([39,40,41,42,43,44,45,46,47,48,49,50,51,52,53,54,55,56]), and (**B**): their subgroup analysis (Human [39,42,47], Rat [40,41,43,44,45,46,48,49,50,51,52,53,54,55,56]).

The pooled effect of soy peptides on weight loss, evaluated across 18 studies, has been depicted in Figure 2A. Overall, soy peptides significantly reduced BW compared with the control group (weighted mean difference (WMD) = −1.96 g, 95% confidence interval [CI]: −3.52, −0.40; *p* < 0.00001), although high heterogeneity (I^2^ = 99%, Cochrane’s Q test, *p* < 0.00001) was noted. Subgroup analysis indicated that soy peptides decreased BW in both humans (WMD = −12.71 g, 95% CI: −30.42, 5.00; *p* < 0.00001) and rats (WMD = −1.73 g, 95% CI: −3.44, −0.03; *p* < 0.00001). The weight-reducing effect reached statistical significance in rats but not in humans (Figure 2B).

### 3.4. Publication Bias

**Figure 3:**
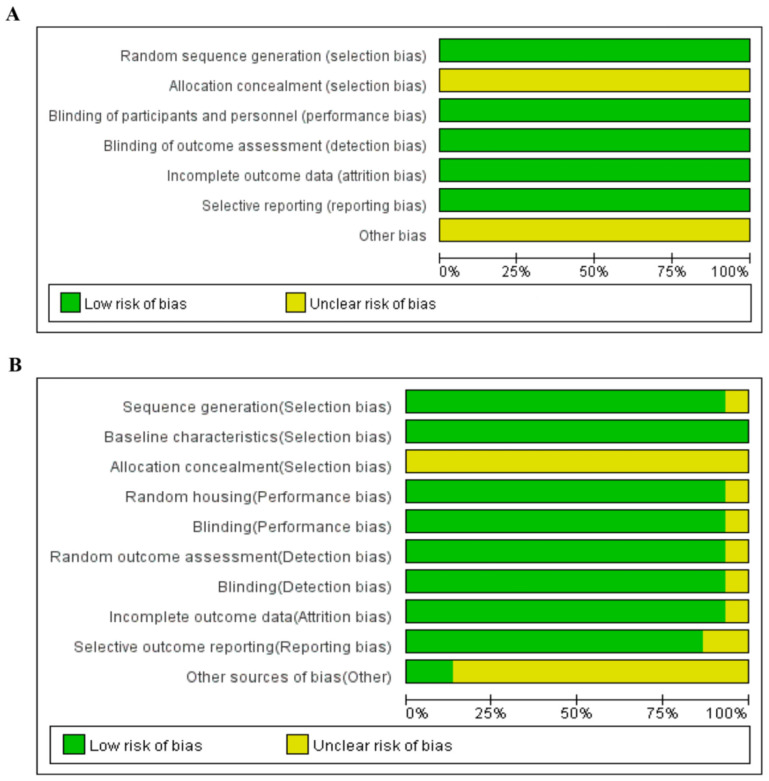
Assessment of risk-of-bias across included human (**A**) and animal (**B**) studies (high risk of bias is not present for human nor animal).

The risk-of-bias assessment for RCTs has been summarized in Figure 3 and Figure S1. Among the 18 included RCTs, the risk of bias was evaluated separately for human and animal studies. The Cochrane and SYRCLE Risk of Bias tools were individually applied for human and animal subjects, respectively. The assessment criteria for animal studies are detailed in Table S1.

In the human trials, three studies were rated “low risk” across the specified domains of random sequence generation (selection bias), blinding of participants and personnel (performance bias), blinding of outcome assessment (detection bias), incomplete outcome data (attrition bias), and selective reporting (reporting bias). Allocation concealment (selection bias) and other sources of bias were rated “unclear” for all studies. No domain emerged with a “high risk” rating among all included studies.

In the animal trials, the baseline characteristics domain (selection bias) was tagged as a “low risk” rating, incorporating all studies (15 studies, 100%). Likewise, most studies were predominantly rated “low risk” for sequence generation (selection bias), random housing (performance bias), blinding (performance bias), random outcome assessment (detection bias), blinding (detection bias), and incomplete outcome data (attrition bias) (14 studies, 93.33%). The domains covering selective outcome reporting (reporting bias) and other sources of bias were also rated as “low risk” (13 studies each, 86.67%). Meanwhile, the allocation concealment domain (selection bias) was rated “unclear,” spanning all studies (15 studies, 100%). The proportion of studies showing a “high risk” rating was zero across all domains.

### 3.5. Sensitivity Analysis

**Figure 4:**
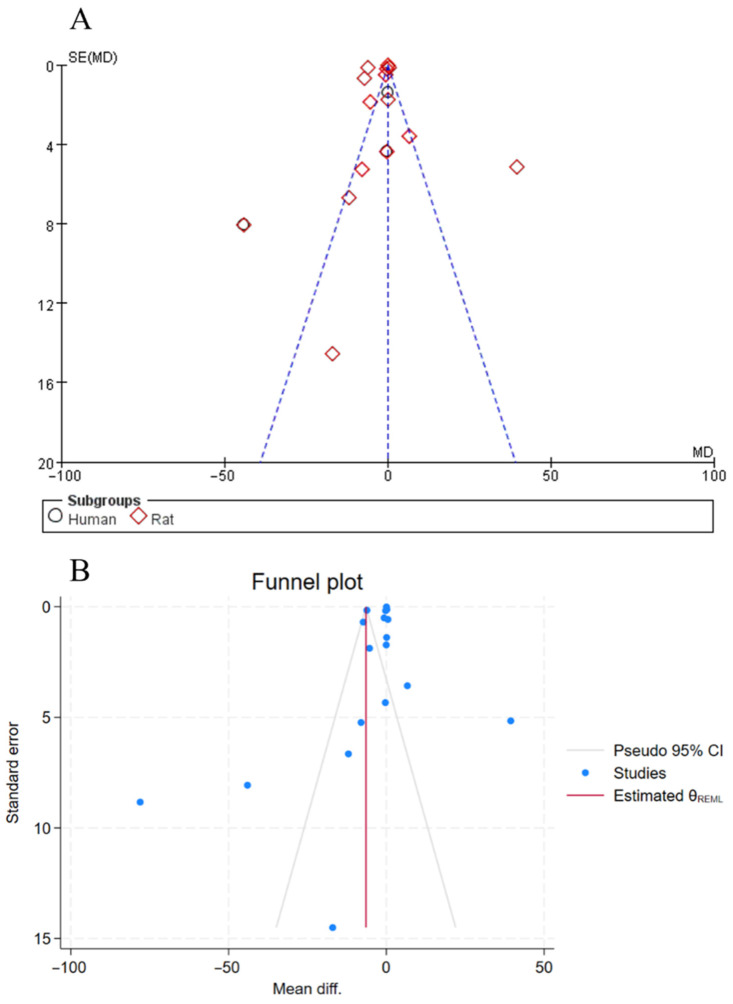
(**A**): Funnel plot illustrating the effect of soy peptides on body weight. (**B**): Funnel plot adjusted using the trim-and-fill method.

For outcome measures with sufficient data (≥10 studies), funnel plots and Egger’s test were employed to formally assess publication bias. As illustrated in Figure 4A, the funnel plots for BW, BWG, and BMI exhibited an approximately symmetrical distribution. However, Egger’s test results indicated statistically significant *p*-values (all *p* < 0.05), suggesting evidence of considerable publication bias. After applying the “trim-and-fill” method, the funnel plots presented a symmetrical pattern (Figure 4B).

## 4. Discussion

To our knowledge, this meta-analysis provides the first comprehensive assessment of the effects of soy peptide interventions on BW in both humans and mice. Direct and indirect evidence synthesized from 18 RCTs revealed that soy peptides exerted a significant impact on BW loss relative to the control group. Subgroup analyses further indicated soy peptide-induced BW reduction in humans and mice. These findings offer nuanced insights into the potential role of soy peptide-containing foods in weight management.

Although a notable weight-reducing effect of soy peptides was observed, the magnitude of this effect varied across the included studies. Several studies reported a decline in BW that did not reach statistical significance. The discrepancy may be attributed to variations in the types of feeds or foods administered to the intervention groups, despite all containing soy peptides. Variations included fermented products, soy protein isolates, and other soy-derived preparations. The intervention duration, ranging from 2 to 26 weeks, may also have influenced the outcomes. Additionally, differences in species or strains of the study subjects contributed to heterogeneous results. For instance, studies involving male Jcl:ICR mice documented non-significant weight reduction, whereas those including male C57BL/6J mice demonstrated a statistically significant decrease in BW.

Soybeans, recognized as a health-beneficial food, have garnered increasing attention for their nutritional composition and weight-loss effects. Recent studies suggest that soy-based foods play a critical role in addressing obesity-related outcomes and hormonal regulation [57,58]. Previous research on weight-loss efficacy typically focused on pharmacological agents, such as drug administration for lowering BW [59,60,61,62,63], which carries potential health and safety risks. Therefore, utilizing soy products as a cleaner alternative to drugs warrants further exploration. Soy products contain various nutritional bioactive components, paving the way for investigating specific constituents crucial to weight reduction. Soy peptides have been associated with cholesterol-lowering and other metabolic benefits, indicative of their plausible involvement in weight management [64]. In our meta-analysis, foods containing soy peptides demonstrated a marked effect on reducing BW. However, due to the complex nutritional profiles of the soy peptide-containing products in the included studies, a comprehensive assessment of whether additional nutritional components contributed to these effects was not achieved, thereby restricting the generalizability of our findings.

Interestingly, intervention groups using fermented soy products with soy peptides (e.g., Cheonggukjang and Doenjang) produced a considerable decrease in BW. In contrast, the use of soy peptide-containing soy protein extracts (e.g., soy crude peptides and soy protein isolate hydrolysate) resulted in a lower BW that did not reach statistical significance. The results suggest that soybean-derived peptides obtained through different methods may variably influence their efficacy for BW reduction.

This study poses several limitations in the interpretation of results. First, most available studies documenting the weight-reducing effects of soy peptides are geographically concentrated (primarily in East Asia and Southeast Asia), with limited representation from other regions, which may affect the comprehensiveness of the findings. Second, variability in study populations—including differential mouse strains and human demographic characteristics—may lead to inconsistencies in experimental outcomes. Third, differences in the food or feed composition provided to intervention groups and trial durations also likely contribute to heterogeneity.

Given these limitations, future studies should include multiple methodological approaches and conduct more extensive research addressing the existing gaps. Comprehensive investigations examining the effects of soy peptides on other obesity-related indicators, such as waist circumference, body fat percentage, and visceral fat area, are needed to strengthen the evidence supporting their weight management efficacy. Furthermore, exploring the signaling pathways involved in soy peptide-mediated weight loss will help elucidate their underlying biological mechanisms of action.

## 5. Conclusions

In summary, the meta-analytic assessment indicates that soy peptides can significantly reduce BW, BWG, and BMI. Subgroup analyses confirm that these effects are evident in both humans and mice. These findings support the potential contribution of soy peptides in modulating BW. For populations at risk of adverse effects from drug-induced weight loss treatments, soy peptide-containing products may represent a feasible alternative. Future research should investigate the direct effects of soy peptide-induced weight reduction and further unveil the underlying mechanisms of their anti-obesity activity.

## Figures and Tables

**Table 1:** Overview of randomized controlled trial studies included in the meta-analysis.

Author, Year	Country	Subjects	RCT	Intervention	Placebo	Duration	Results
Akiko Taniguchi-Fukatsu, 2012 [39]	Japan	Human	Randomized, controlled	Test meal	Control meal	8 weeks	Body weight decreased
Chung Shil Kwak, 2012 [40]	Republic of Korea	Male Sprague–Dawley rats	Randomized, controlled	Doenjang	Steamed soybeans	8 weeks	Body weight gain decreased
Dae Young Kwon, 2009 [41]	Republic of Korea	Male Sprague-Dawley rats	Randomized, controlled	Kochujang	Control diet	3 weeks	Body weight decreased significantly
Diana Nur Afifah, 2020 [42]	Indonesia	Women aged 30 years or older	Randomized pre–post-test control group design	Tempeh gembus	Control diet	2 weeks	Body weight decreased
Dong-Ju Kim, 2008 [43]	Republic of Korea	Male C57BL/KsJ-db/dbmice	Randomized, controlled	Chungkukjang	Standard semisynthetic diet	6 weeks	Body weight decreased significantly
Hayoung Woo, 2023 [44]	Republic of Korea	Male Sprague-Dawley rats	Randomized, controlled	Doenjang	High fat diet	13 weeks	Significant reduction of body weight
Jiyoung Kim, 2017 [45]	Republic of Korea	Male C57BL/6J mice	Randomized, controlled	Cheonggukjang	Control diet	26 weeks	Body weight gain decreased significantly
JooHee Choi, 2016 [46]	Republic of Korea	Male C57BL/6J mice	Randomized, controlled	Cheonggukjang	Control diet	11 weeks	Body weight gain decreased significantly
Jung Hyun Kwak, 2012 [47]	Republic of Korea	Human	Randomized, controlled	Black soy peptide (BSP)	Casein	12 weeks	BMI decreased
Kengo Ishihara, 2002 [48]	Japan	Male KK-Ay mice	Randomized, controlled	Soy protein isolate hydrolysate (SPI-H)	Casein	4 weeks	Body weight decreased
Luthfia Dewi, 2020 [49]	Indonesia	Sprague Dawley male rats	Randomized, controlled	Tempeh	Normal diet/High cholesterol diet	3 weeks	Body weight significantly lower than control groups
Motoi Tamura, 2024 [50]	Japan	Male Jcl:ICR mice	Randomized, controlled	Natto	Control diet	6 weeks	Body weight decreased
Nao Inoue, 2011 [51]	Japan	Male OLETF rats	Randomized, controlled	Soy crude peptides (SCP)	Control diet	4 weeks	Body weight decreased
Na Young Park, 2012 [52]	Republic of Korea	Male C57BL/6J mice	Randomized, controlled	Brown rice doenjang	High fat diet	8 weeks	Body weight gain decreased significantly
Satoshi Wanezaki, 2020 [53]	Japan	Male OLETF and Long-Evans Tokushima Otsuka (LETO) wild-type rats	Randomized, controlled	Soy β-conglycinin (βCG)	Control diet	4 weeks	Body weight gain decreased
Shizuka TAMARU, 2007 [54]	Japan	Male Wistar rats	Randomized, controlled	Soybean protein isolate (SPI)	Casein	4 weeks	Body weight gain decreased
Yuko Yamada, 2012 [55]	Japan	Male KKAy mice	Randomized, controlled	Soymorphin-5	Control diet	6 weeks	Body weight showed no differences between control and Soymorphin-5
Yun Jung Kim, 2008 [56]	Republic of Korea	Male Sprague-Dawley rats	Randomized, controlled	The mixture-supplemented HFD group	High-fat control diet	9 weeks	Body weight gain decreased significantly

## Data Availability

The original contributions presented in the study are included in the article, further inquiries can be directed to the corresponding author.

## Supplementary Materials

The following supporting information can be downloaded at: https://www.mdpi.com/article/10.3390/foods15071191/s1, Figure S1: Graph of risk bias assessment included human (A) and animal (B) studies, Table S1: SYRCLE’s tool for assessing risk of bias.
