# Supplementary material for: Anti-Obesity Effects of Soy Peptides In Vivo: A Meta-Analysis of Randomized Controlled Trials
Source: Foods. 2026 Apr 1;15(7):1191. doi: 10.3390/foods15071191 (PMC13073697; doi:10.3390/foods15071191)
Supplement: Supplementary file 1 [file foods-15-01191-s001.zip › foods-4128273-supplementary.pdf]

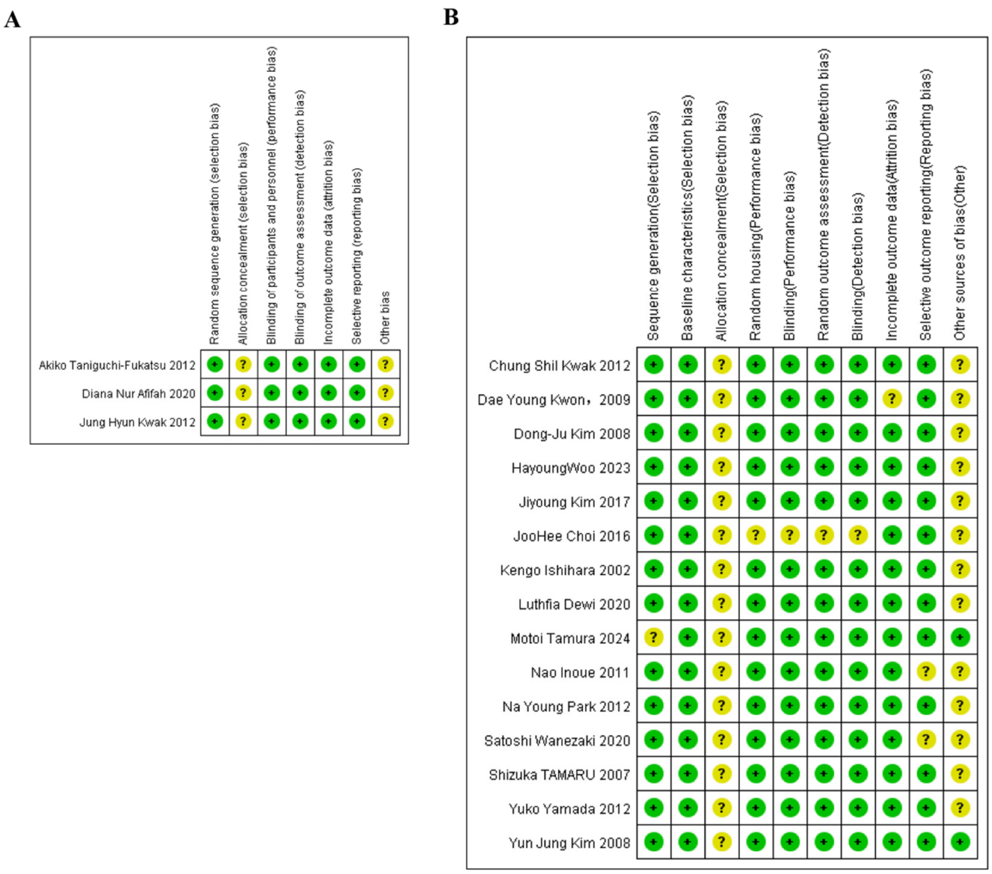

Figure S1. Graph of risk bias assessment included human (A) [39,42,47] and animal (B) studies [40–41,43–46,48-56].

Table S1. SYRCLE's tool for assessing risk of bias

Item	Type of bias	Domain	Description of domain	Review authors judgment	Answer question	Risk of bias
1	Selection bias	Sequence generation	Describe the methods used, if any, to generate the allocation sequence in sufficient detail to allow an assessment whether it should produce comparable groups.	Was the allocation sequence adequately generated and applied? (*)	Yes/No/Unclear	Low/High/Unclear
2	Selection bias	Baseline characteristics	Describe all the possible prognostic factors or animal characteristics, if any, that are compared in order to judge whether or not intervention and control groups were similar at the start of the experiment.	Were the groups similar at baseline or were they adjusted for confounders in the analysis?	Yes/No/Unclear	Low/High/Unclear
3	Selection bias	Allocation concealment	Describe the method used to conceal the allocation sequence in sufficient detail to determine whether intervention allocations could have been foreseen before or during enrolment.	Was the allocation adequately concealed? (*)	Yes/No/Unclear	Low/High/Unclear
4	Performance bias	Random housing	Describe all measures used, if any, to house the animals randomly within the animal room.	Were the animals randomly housed during the experiment?	Yes/No/Unclear	Low/High/Unclear
5	Performance bias	Blinding	Describe all measures used, if any, to blind trial caregivers and researchers from knowing which intervention each animal received. Provide any information relating to whether the intended blinding was effective.	Were the caregivers and/or investigators blinded from knowledge which intervention each animal received during the experiment?	Yes/No/Unclear	Low/High/Unclear
6	Detection bias	Random outcome assessment	Describe whether or not animals were selected at random for outcome assessment, and which methods to select the animals, if any, were	Were animals selected at random for outcome assessment?	Yes/No/Unclear	Low/High/Unclear

			used.			
7	Detection bias	Blinding	Describe all measures used, if any, to blind outcome assessors from knowing which intervention each animal received. Provide any information relating to whether the intended blinding was effective.	Was the outcome assessor blinded?	Yes/No/Unclear	Low/High/Unclear
8	Attrition bias	Incomplete outcome data	Describe the completeness of outcome data for each main outcome, including attrition and exclusions from the analysis. State Whether attrition and exclusions were reported, the numbers in each intervention group (compared with total randomized animals), reasons for attrition or exclusions, and any re-inclusions in analyses for the review.	Were incomplete outcome data adequately addressed? (*)	Yes/No/Unclear	Low/High/Unclear
9	Reporting bias	Selective outcome reporting	State how selective outcome reporting was examined and what was found.	Are reports of the study free of selective outcome reporting? (*)	Yes/No/Unclear	Low/High/Unclear
10	Other	Other sources of bias	State any important concerns about bias not covered by other domains in the tool.	Was the study apparently free of other problems that could result in high risk of bias? (*)	Yes/No/Unclear	Low/High/Unclear

* Items in agreement with the items in the Cochrane Risk of Bias tool.

39.Taniguchi-Fukatsu, A.; Yamanaka-Okumura, H.; Naniwa-Kuroki, Y.; Nishida, Y.; Yamamoto, H.; Taketani, Y.; Takeda, E. Natto and viscous vegetables in a Japanese-style breakfast improved insulin sensitivity, lipid metabolism and oxidative stress in overweight subjects with impaired glucose tolerance. *Brit. J. Nutr.* **2012**, *107*, 1184–1191

40.Kwak, C.S.; Park, S.C.; Song, K.Y. *Doenjang*, a fermented soybean paste, decreased visceral fat accumulation and adipocyte size in rats fed with high fat diet more effectively than nonfermented soybeans. *J. Med. Food* **2012**, *15*, 1–9.

41.Kwon, D.Y.; Hong, S.M.; Ahn, I.S.; Kim, Y.S.; Shin, D.W.; Park, S. *Kochujang*, a Korean fermented red pepper plus soybean paste, improves glucose homeostasis in 90% pancreatectomized diabetic rats. *Nutrition* **2009**, *25*, 790–799.
43.Kim, D.J.; Jeong, Y.J.; Kwon, J.H.; Moon, K.D.; Kim, H.J.; Jeon, S.M.; Lee, M.K.; Park, Y.B.; Choi, M.S. Beneficial effect of chungkukjang on regulating blood glucose and pancreatic β-cell functions in C75BL/KsJ-*db*/*db* mice. *J. Med. Food* **2008**, *2*, 215–223.
44.Woo, H.; Han, A.; Park, J.E.; Cha, Y.S. Korean fermented soybean paste (*Doenjang*) has anti-obesity and anti-hypertensive effects via the renin-angiotensin system (RAS) in high-fat diet-induced obese rats. *PLoS ONE* **2023**, *18*, e0291762.
45.Kim, J. Anti-obesity and Anti-inflammation Effects of Cheonggukjang in C57Bl/6 mice with High Fat Diet Induced Obesity. *J. Life Sci.* **2017**, *27*, 1357–1368.
46.Choi, J.H.; Pichiah, P.B.; Kim, M.J.; Cha, Y.S. *Cheonggukjang*, a soybean paste fermented with *B. licheniformis*-67 prevents weight gain and improves glycemic control in high fat diet induced obese mice. *J. Clin. Biochem. Nutr.* **2016**, *59*, 31–38.
48.Ishihara, K.; Fukuchi, Y.; Segawa, K.; Takahashi, M.; Mita, Y.; Fukuya, Y.; Yasumoto, K.; Oyaizu, S.; Mizunoya, W.; Fushiki, T. A soybean peptide isolate diet promotes postprandial carbohydrate oxidation and energy expenditure in type II diabetic mice. *J. Nutr.* **2003**, *133*, 752–757.
49.Dewi, L.; Lestari, L.A.; Astiningrum, A.N.; Fadhila, V.; Amala, N.; Bakrie, M.A.; Hidayah, N. Tempeh and red ginger flour for hypercholesterolemic rats. *Nutr. Food Sci.* **2021**, *51*, 41–49.
50.Tamura, M.; Watanabe, J.; Noguchi, T.; Nishikawa, T. High poly-γ-glutamic acid-containing natto improves lipid metabolism and alters intestinal microbiota in mice fed a high-fat diet. *J. Clin. Biochem. Nutr.* **2024**, *74*, 47–56.
51.Inoue, N.; Nagao, K.; Sakata, K.; Yamano, N.; Gunawardena, P.E.; Han, S.Y.; Matsui, T.; Nakamori, T.; Furuta, H.; Takamatsu, K.; et al. Screening of soy protein-derived hypotriglyceridemic di-peptides in vitro and in vivo. *Lipids Health Dis.* **2011**, *10*, 85.
52.Park, N.Y.; Rico, C.W.; Lee, S.C.; Kang, M.Y. Comparative effects of doenjang prepared from soybean and brown rice on the body weight and lipid metabolism in high fat-fed mice. *J. Clin. Biochem. Nutr.* **2012**, *51*, 235–340.
53.Wanezaki, S.; Saito, S.; Inoue, N.; Tachibana, N.; Shirouchi, B.; Sato, M.; Yanagita, T.; Nagao, K. Soy β-conglycinin peptide attenuates obesity and lipid abnormalities in obese model OLETF rats. *J. Oleo Sci.* **2020**, *69*, 495–502.
54.Tamaru, S.; Kurayama, T.; Sakono, M.; Fukuda, N.; Nakamori, T.; Furuta, H.; Tanaka, K.; Sugano, M. Effects of dietary soybean peptides on hepatic production of ketone bodies and secretion of triglyceride by perfused rat liver. *Biosci. Biotechnol. Biochem.* **2007**, *71*, 2451–2457.
55.Yamada, Y.; Muraki, A.; Oie, M.; Kanegawa, N.; Oda, A.; Sawashi, Y.; Kaneko, K.; Yoshikawa, M.; Goto, T.; Takahashi, N.; et al. Soymorphin-5, a soy-derived μ-opioid peptide, decreases glucose and triglyceride levels through activating adiponectin and PPARα systems in diabetic KKA^y mice. *Am. J. Physiol. Endocrinol. Metab.* **2012**, *302*, E433–E440.
56.Kim, Y.J.; Kim, K.Y.; Kim, M.S.; Lee, J.H.; Lee, K.P.; Park, T. A mixture of the aqueous extract of Garcinia cambogia, soy peptide and L: -carnitine reduces the accumulation of visceral fat mass in rats rendered obese by a high fat diet. *Genes Nutr.* **2008**, *2*, 353–358.
